# Research Recommendations for Selected IARC-Classified Agents

**DOI:** 10.1289/ehp.0901828

**Published:** 2010-06-18

**Authors:** Elizabeth M. Ward, Paul A. Schulte, Kurt Straif, Nancy B. Hopf, Jane C. Caldwell, Tania Carreón, David M. DeMarini, Bruce A. Fowler, Bernard D. Goldstein, Kari Hemminki, Cynthia J. Hines, Kirsti Husgafvel Pursiainen, Eileen Kuempel, Joellen Lewtas, Ruth M. Lunn, Elsebeth Lynge, Damien M. McElvenny, Hartwig Muhle, Tamie Nakajima, Larry W. Robertson, Nathaniel Rothman, Avima M. Ruder, Mary K. Schubauer-Berigan, Jack Siemiatycki, Debra Silverman, Martyn T. Smith, Tom Sorahan, Kyle Steenland, Richard G. Stevens, Paolo Vineis, Shelia Hoar Zahm, Lauren Zeise, Vincent J. Cogliano

**Affiliations:** 1 American Cancer Society, Atlanta Georgia, USA; 2 National Institute for Occupational Safety and Health, Cincinnati, Ohio, USA; 3 International Agency for Research on Cancer, Lyon, France; 4 Institute universitaire romand de Sante au Travail, Lausanne, Switzerland; 5 U.S. Environmental Protection Agency, Research Triangle Park, North Carolina, USA; 6 Agency for Toxic Substances and Disease Registry, Chamblee, Georgia, USA; 7 University of Pittsburgh, Pittsburgh, Pennsylvania, USA; 8 German Cancer Research Center, Heidelberg, Germany; 9 Finnish Institute of Occupational Health, Helsinki, Finland; 10 University of Washington, Seattle, Washington, USA; 11 National Institute of Environmental Health Sciences, National Institutes of Health, Department of Health and Human Services, Research Triangle Park, North Carolina, USA; 12 University of Copenhagen, Copenhagen, Denmark; 13 University of Central Lancashire/Westlakes Scientific Consulting, Cumbria, Lancashire, United Kingdom; 14 Fraunhofer Institute of Toxicology and Experimental Medicine, Hannover, Germany; 15 Nagoya University Graduate School of Medicine, Nagoya, Japan; 16 University of Iowa, Iowa City, Iowa, USA; 17 National Cancer Institute, National Institutes of Health, Department of Health and Human Services, Bethesda, Maryland, USA; 18 University of Montreal Hospital Research Center, Montreal, Quebec, Canada; 19 University of California, Berkeley, Berkeley, California, USA; 20 Institute of Occupational and Environmental Medicine, University of Birmingham, Birmingham, United Kingdom; 21 Rollins School of Public Health, Emory University, Atlanta, Georgia, USA; 22 University of Connecticut Health Center, Farmington, Connecticut, USA; 23 Imperial College London, London, United Kingdom; 24 California Environmental Protection Agency, Oakland, California, USA

**Keywords:** animal, carcinogen, carcinogenesis, epidemiology, human, IARC, mechanisms of carcinogenicity, occupational

## Abstract

**Objectives:**

There are some common occupational agents and exposure circumstances for which evidence of carcinogenicity is substantial but not yet conclusive for humans. Our objectives were to identify research gaps and needs for 20 agents prioritized for review based on evidence of widespread human exposures and potential carcinogenicity in animals or humans.

**Data sources:**

For each chemical agent (or category of agents), a systematic review was conducted of new data published since the most recent pertinent International Agency for Research on Cancer (IARC) Monograph meeting on that agent.

**Data extraction:**

Reviewers were charged with identifying data gaps and general and specific approaches to address them, focusing on research that would be important in resolving classification uncertainties. An expert meeting brought reviewers together to discuss each agent and the identified data gaps and approaches.

**Data synthesis:**

Several overarching issues were identified that pertained to multiple agents; these included the importance of recognizing that carcinogenic agents can act through multiple toxicity pathways and mechanisms, including epigenetic mechanisms, oxidative stress, and immuno- and hormonal modulation.

**Conclusions:**

Studies in occupational populations provide important opportunities to understand the mechanisms through which exogenous agents cause cancer and intervene to prevent human exposure and/or prevent or detect cancer among those already exposed. Scientific developments are likely to increase the challenges and complexities of carcinogen testing and evaluation in the future, and epidemiologic studies will be particularly critical to inform carcinogen classification and risk assessment processes.

Forty-five years after the World Health Organization recognized cancer as a world health problem by creating the International Agency for Research on Cancer (IARC), carcinogenic exposures in the workplace remain a concern. Many known and suspected carcinogens are found in today’s workplaces, and uncertainties about the health effects of exposure to these hazards have delayed regulatory action and the search for safer alternatives. In this review we focus primarily on chemicals, metals, dusts, and physical agents for which there is widespread human exposure, predominantly in occupational settings, and we address unresolved questions regarding carcinogenicity. Most of these agents are in IARC Groups 2A and 2B—agents for which there is sufficient evidence of carcinogenicity in animals but limited evidence for carcinogenicity in humans.

A project to systematically identify data gaps was initiated by the National Occupational Research Agenda team of the U.S. National Institute for Occupational Safety and Health (NIOSH) to enhance occupational cancer research and involved joint planning with IARC, the American Cancer Society, the U.S. National Institute of Environmental Health Sciences, and the U.S. National Cancer Institute. In this review we present the results of this effort and identify opportunities for further research that would resolve classification uncertainties for selected high-priority agents. The process included a meeting to identify high-priority agents; expert reviews of each agent to update the literature since the last Monograph evaluation and to identify research priorities; and a workshop to discuss the identified data gaps and approaches. Expert reviewers were selected by the planning committee based on expertise in epidemiology and toxicology and on knowledge of the agents. For many agents, we recognized that opportunities for cohort studies would be limited, and reviewers were encouraged to consider possible experimental studies to elucidate carcinogenic mechanisms and molecular epidemiologic studies to develop intermediate biomarker data that could be used in classification.

Full reviews and recommendations will be published in an IARC technical report. Here, we summarize recommendations for each of the agents and address some overarching topics pertaining to several agents or categories of agents.

## Overarching Topics

### Carcinogenic mechanisms

Most tumors arise from multiple genetic and epigenetic changes, many of which are difficult to measure *in vivo* in experimental animals or humans. Genetic changes can be broadly defined to involve either inherited or somatic changes in the DNA sequence. Epigenetic modifications generally involve modification (e.g., by methylation or acetylation) of DNA or histones in chromatin or the binding of microRNAs (noncoding RNAs 21–23 bases) to homologous sequences in mRNA, resulting in a double-stranded structure that can decrease production of the corresponding protein (Garzon et al. 2009; Mathews et al. 2009). Recent advances in cancer biology support the view that carcinogenic agents can act through multiple toxicity pathways and mechanisms, including both genetic and epigenetic changes. Alterations in gene expression and levels of key proteins are considered an essential component of the mechanisms by which most tumors arise (Croce 2009; Jones and Baylin 2007). Although standard methods for detecting agents that cause mutation have been in place for decades, no standardized, validated assays are available for routine assessments for epigenetic events.

“Omics,” the study of large sets of biological molecules, is an emerging tool to study genetic and epigenetic events related to specific exposures. Although the number of omic techniques is ever expanding, the most developed techniques are high-throughput DNA sequencing, transcriptomics (studying gene expression), epigenomics (studying epigenetic regulation of gene expression), proteomics (studying large sets of proteins; the proteome), and metabolomics (studying large sets of metabolites; the metabolome). Omic technologies can be used to study the effects of the same chemicals in experimental animals and in human cells *in vitro,* eventually allowing for a more comprehensive human carcinogenicity and assessment of carcinogenic mechanisms. A broad all-encompassing approach is needed that uses the same technologies in experimental animals, human cells in culture, and human populations. Eventually, a bioinformatics database of human responses to different chemical exposures and associated chronic diseases could be used to compare the effects of novel chemicals with those of established carcinogens. Given the sensitivity of omic analyses, low-dose adverse effects could also be observed and distinguished from high-dose phenomena, and if exposures were accurately assessed, dose–response data could be incorporated into risk assessments.

Oxidative stress has been invoked as a mechanism in the carcinogenicity of a number of agents, including some metals and particles. Oxidative damage to cellular DNA, the epigenome (including proteins), and lipids can occur when reactive oxygen species (ROS) escape cell antioxidant and repair mechanisms (Mayne 2003; Shi et al. 2004; Valavanidis et al. 2009). Proposed carcinogenic mechanisms include direct genotoxicity as well as tumor promotion (e.g., arsenic and perhaps other metals are thought to promote tumors by causing oxidative stress that interferes with apoptosis) (Shi et al. 2004). Several methodologic issues present challenges to validation of oxidative stress biomarker assays, including highly variable background levels of specific DNA lesions (e.g., between individuals and between experiments) and the need to consider biomarkers of nitration as well as oxidation (Mayne 2003). Research is needed to examine the relationship between exposure to toxic agents and oxidative stress biomarkers and between these biomarkers and risk of cancer, while controlling for the many individual factors that contribute to oxidative stress. Guidelines on standardizing the collection and measurement of oxidative stress biomarkers in humans (American Thoracic Society 1999; Horvath et al. 2005) are important to facilitate their effective use.

Immunomodulation is also associated with cancer in humans and plays a particularly important role for some lymphomas (Hartge et al. 2006) and other cancers. Although biological markers measured in blood are available to assess clinically significant immune dysfunction, identification and standardization of biomarkers of more subtle changes in immune status in humans with specific exposures is complicated by the enormous variety of markers and assays and the high level of intraindividual and interindividual variability due, in part, to the inherently dynamic role of the immune system. Several agents discussed here [e.g., polychlorinated biphenyls (PCBs)] have been associated with lymphomas, and investigation of their immunomodulatory effects may clarify their carcinogenic potential.

Addressing the role of genetic susceptibility to carcinogenic exposures is also important; however, the stable and reproducible associations are few. Examining genetic polymorphisms related to carcinogen metabolism and/or DNA repair may facilitate identification of higher cancer risks in susceptible subgroups and clarify the role of specific agents in mixed exposures. However, the magnitude of such associations may be modest and involve multiple genes or metabolic pathways, making them difficult to detect.

### Issues in exposure assessment

Some agents considered in this review may occur as extremely small particles with at least one dimension between 1 and 100 per unit mass than larger particles of the same composition, and the smaller particles appear to be more biologically reactive, toxic, and carcinogenic than larger-size particles. Thus, their toxic effects may need to be evaluated separately from larger particles of the same chemical composition (Schulte et al. 2009). Critical exposure metrics that should be included are particle count, surface area, mass, and density. Other physical and chemical properties can influence the biological activity and toxicity of nanoparticles, including contaminants and the degree of agglomeration (Schulte et al. 2009). Use of several agents we consider to be nanoparticles is increasing, including nano-titanium dioxide (TiO_2_), in products.

In occupational settings where many Group 2 carcinogens are used, levels of exposure may be relatively low and potential for multiple exposures high. High-quality exposure assessment will be required to assess quantitative exposure response for specific agents while accounting for other potentially carcinogenic exposures. Historical monitoring data, when available, may be used to create a job-exposure matrix. Biomarkers of exposure to agents with long biological half-lives, such as serum levels of PCBs (Burns et al. 2008), may be useful in assessing historical exposures, whereas biomarkers of internal dose, such as hemoglobin adducts, may be useful to characterize recent exposures (Angerer et al. 2007). Biomarkers of effects related to carcinogenicity, such as DNA adducts in urothelial cells (Zhou et al. 1997) and chromosomal aberrations in peripheral lymphocytes (Yong et al. 2009), are useful when population size, latency, and/or lack of historical data preclude study of traditional epidemiologic end points.

### Study design

Some agents discussed here are used primarily in small businesses with high turnover, where it is difficult to assemble large study populations. Alternative approaches include use of union records, national census records, or records of individuals licensed to perform certain work (e.g., certified pesticide applicators), case–control studies with enhanced exposure assessment, and cross-sectional surveys examining intermediate markers. It may be possible to recruit participants outside of the workplace through the media and then use a validated biomarker to confirm and quantify recent exposure.

Epidemiologic studies using death certificates may fail to identify excesses in cancer sites with high survival rates or excesses in specific morphologic types of cancer. The ability to study cancer incidence rather than mortality—linking occupational cohorts with regional or national cancer registries—would improve detection of cancers with high survival rates, the accuracy of diagnostic information, and more timely identification of carcinogenic hazards. In several Nordic countries, national cancer registries that are linked to census occupational data (and in Norway to a serum bank) have been an important resource for studies of occupational and environmental exposures. Such resources could be developed in other countries.

Interpretation of evidence for excesses in lymphohematopoietic cancers (LHC) for several agents has been complicated by inconsistencies in specific tumor sites. These differences may result from inaccuracy of death certificate diagnosis as well as from changes in LHC classification and grouping over time. Epidemiologic and animal studies may consider morphologically distinct hematologic cancers as separate end points, even though they may share common cellular origins. As knowledge of hematologic malignancies evolves, it is important to reexamine approaches to disease categorization in epidemiology and animal toxicology. Over time, there has been growing recognition of close relationships and overlap of such morphologically diverse disorders as chronic lymphocytic leukemia and multiple myeloma, now considered subclassifications of mature B-cell neoplasms (Swerdlow et al. 2008).

### Data gaps and research priorities for specific agents

The agents, exposure circumstances, and prior IARC Monograph evaluations of the agents considered are listed in the Supplemental Material (doi:10.1289/ehp.0901828).

#### Lead and lead compounds

Although the occurrence of lead in the environment has decreased greatly because of the elimination of most leaded gasoline, substantial occupational exposures continue primarily via lead in the battery industry and lead pigments in paints (IARC 2006c). Evidence for carcinogenicity in workers exposed to inorganic lead is most consistent for stomach cancer (rate ratio, 1.3–1.5), with lung, kidney, and brain cancer showing elevation in some but not all studies (IARC 2006c). Background rates of stomach cancer are highly variable; therefore, epidemiologic studies should consider local referent rates and internal dose–response analyses. Additional studies of new cohorts with well-documented lead exposure, as well as further follow-up of existing cohorts, would be useful. A study of the NIOSH Adult Blood Lead Exposure Surveillance (ABLES) registry—which includes 50,000 workers with at least one blood lead measurement during 1990–2007—is currently under way. Future studies could be strengthened by including *a*) assessment of the correlation of blood lead measurements with cumulative exposure as measured by bone lead; *b*) assessment of whether *Helicobacter pylori* infection is associated with higher blood lead levels; and *c*) evaluation of genetic susceptibility factors, such as polymorphisms in the δ-aminolevulinate dehydratase (*ALAD*) gene. Further experimental research is needed to evaluate the mechanisms by which lead may cause cancer, with particular emphasis on oxidative stress/apoptosis and the roles of cellular defense mechanisms, signaling pathways, and intracellular lead-binding patterns.

#### Indium phosphide and other indium compounds

Intratracheal installation of indium phosphide causes pulmonary inflammation and high incidences of lung tumors in experimental animals (IARC 2006b). No epidemiologic studies have evaluated indium compounds specifically for cancer. Studies of workers in the U.S. semiconductor industry are unlikely to be informative because of limited historical exposure, multiple exposures in wafer fabrication, and little historical exposure-monitoring information. Epidemiologic studies, if feasible, may be most informative in secondary indium-refining industries (primary refining likely results in lower indium and higher cadmium exposure). Recent findings of pulmonary effects among indium workers in Asia (Chonan et al. 2007; Hamaguchi et al. 2008) should be investigated further. Concurrent studies of exposure and biomarkers of genetic damage, such as chromosomal aberrations in accessible cells of exposed workers (e.g., nasal epithelium, buccal cells, shed urinary cells, or circulating lymphocytes), may be useful. Further experimental research should investigate mechanisms of indium compound–induced toxicity and carcinogenicity, with particular focus on oxidative stress, inhibition of protective protein synthetic mechanisms, and DNA damage.

#### Metallic cobalt (with or without tungsten carbide)

The evidence for carcinogenicity of cobalt with tungsten carbide in humans comes from studies finding increased lung cancer risks among workers in the hard-metal industry in France and Sweden (IARC 2006d). The prevalence of such exposures is increasing (Busch et al. 2010). There is good experimental evidence that cobalt and cobalt with tungsten carbide produce cellular toxicity via formation of ROS, leading to oxidative stress and triggering a number of cellular regulatory pathways (Fenoglio et al. 2008). Research recommendations include updating the French and Swedish studies and studying additional cohorts of hard-metal manufacturing workers; these studies should include assessment of molecular biomarkers of early cellular effects and genetic polymorphisms associated with cellular protective systems. Further research is needed into the toxicity of exposure to cobalt with tungsten carbide in the nanoparticle size range.

#### Welding

Epidemiologic studies indicate a 20–40% increased risk of lung cancer among welders (Ambroise et al. 2006; Siew et al. 2008). Experimental studies are suggestive—but not conclusive—of lung carcinogenicity of welding-fume exposure (Antonini 2003; Zeidler-Erdely et al. 2008). Many *in vitro* and *in vivo* studies have shown welding fumes to be genotoxic (Antonini et al. 2003). Pulmonary effects consistent with oxidative stress and inflammatory responses have been observed in experimental animals. Genotoxic effects observed in welders include elevated 8-hydroxydeoxyguanine in urine; DNA–protein crosslinks, sister chromatid exchanges, and increased micronuclei in lymphocytes; increased DNA strand breaks, chromosome aberrations, and increased micronuclei in buccal epithelial cells (Antonini et al. 2003; Danadevi et al. 2004). Research needs include reexamination of existing cohorts and establishing new cohorts with improved exposure assessment (e.g., the type of welding process, the type of metal being welded, the types of rods and fluxes used, and other characteristics of the welding environment such as abrasives, cleaners, and degreasers used, and if feasible, biomarkers of exposure to manganese or iron) and improved smoking data. Experimental studies are needed on inhalation exposure to different types of welding fumes, including ultrafine/nano-size particles, and on epigenetic mechanisms, gene expression pathways, and functional level changes related to welding fume exposure (Rim et al. 2007; Salnikow and Zhitkovich 2008). In addition, welders have an increased risk of ocular melanoma (El Ghissassi et al. 2009). Further research is needed to determine whether this is due to ultraviolet radiation, other forms of electromagnetic radiation, or metal and chemical fumes emitted during welding.

#### TiO_2_

Elevated lung tumor rates have been observed in rats after chronic inhalation or intratracheal administration of TiO_2_ (Baan et al. 2006). A consistent dose–response relationship for either pulmonary inflammation or lung tumor response was observed for fine and ultrafine TiO_2_ particle sizes when dose was expressed as the particle surface area retained in rat lungs (Dankovic et al. 2007). These data include doses associated with the overloading of rat lung particle clearance, which occurs at lower mass doses for ultrafine TiO_2_ than for fine-sized TiO_2_, and is related to the increased surface area of the ultrafine particles. Lung overload is associated with persistent pulmonary inflammation, ROS, cell injury and proliferation, and fibrosis in rats and mice; and with gene mutation and lung tumors in rats. Qualitatively similar lung responses, including reduced lung clearance, pulmonary inflammation, and fibrosis, have been observed in workers in dusty jobs, although elevated lung tumors have not been observed in epidemiologic studies of TiO_2_ workers (Baan et al. 2006).

Recent subchronic studies in rats confirm earlier findings that particle size (as well as crystal structure) and coatings can influence pulmonary responses (inflammation, cytotoxicity, and cell proliferation) to TiO_2_ (Sager et al. 2008; Sager and Castranova 2009; Warheit et al. 2006, 2007) and suggest that inhaled TiO_2_ may act through a secondary genotoxic mechanism involving chronic inflammation and oxidative stress related to particle surface area (Schins and Knaapen 2007). The observation of inhaled discrete nanoscale TiO_2_ particles inside rat alveolar epithelial cell organelles, including the nucleus (Geiser et al. 2005), suggests that direct genotoxic mechanisms are also possible (Schins and Knaapen 2007). Epidemiologic studies with well-characterized exposures and adequate follow-up are needed, especially for workers producing or using nanoscale TiO_2_. Possible cohorts include workers in industries using nanoscale TiO_2_, such as the cosmetic industry. Given increasing applications of nano-TiO_2_ in consumer products, there is a need to develop better techniques to detect TiO_2_ in tissues and to examine possible carcinogenicity of nano-TiO_2_ by other routes of exposure (e.g., oral, dermal).

#### Diesel engine exhaust

Two meta-analyses estimated the summary risk for lung cancer and diesel engine exhaust (DE) exposure to range from 1.33 [95% confidence interval (CI), 1.24–1.44] (Bhatia et al. 1998) to 1.47 (95% CI, 1.29–1.67) (Lipsett and Campleman 1999); only a few studies have included retrospective exposure assessment (Garshick et al. 2008; Neumeyer-Gromen et al. 2009; Steenland et al. 1990). Two studies nearing completion will provide information on quantitative exposure–response data based on historical exposure estimates. These include a cohort and nested case–control study of lung cancer in U.S. nonmetal miners with a wide range of DE exposure (National Research Council and Institute of Medicine 2008) and additional retrospective exposure assessment in a truck driver cohort with light-to-moderate DE exposure (Garshick E, personal communication). If the research demonstrates exposure response, it will be important to identify the underlying mechanisms of DE-induced carcinogenesis and identify the components of DE that are most biologically active in humans. DNA adducts formed by nitro-polycyclic aromatic hydrocarbons (PAHs) and PAHs in animal and cellular studies have been well documented. These and other biomarkers could be incorporated in cross-sectional epidemiologic studies of DE exposure and biomarkers of inflammation, genotoxicity, and other relevant early biological effects.

#### Refractory ceramic fibers

Refractory ceramic fibers (RCF), which have replaced asbestos as high-temperature insulation, induce benign and malignant lung tumors in rats (Mast et al. 1995). Only one small U.S. occupational cohort exposed to these biopersistent fibers has been studied; at last follow-up, there were only nine lung cancer deaths (LeMasters et al. 2003). A European study found an exposure-related excess of pleural plaques after controlling for past asbestos exposure (Cowie et al. 2001). Identification and follow-up of new and established U.S. and European cohorts would be useful. Animal research has not been conducted on the combined effects of RCF and granular, low-biosoluble particles such as TiO_2_, which can aggravate effects of inhaled fibers. The impact of fiber length on carcinogenicity should also be investigated.

The validity of negative dose–response data in rats after inhalation exposure to RCF is questionable because there are indications that the sensitivity of the rat inhalation model with man-made fibers is relatively low (Muhle and Pott 2000; Wardenbach et al. 2005). Future research in developing a sensitive rat inhalation model for RCF is needed.

#### Carbon black

Sorahan and Harrington (2007) reported elevated lung cancer in an update of the U.K. carbon worker cohort standardized mortality ratio, 1.46; 95% CI, 1.13–1.85), with some analyses suggesting that carbon black may be a late-stage carcinogen. No new chronic studies in animals have been published since the IARC Monograph (Baan et al. 2006). Several recent subchronic studies in rats and mice (Duffin et al. 2007; Sager and Castranova 2009; Stoeger et al. 2006) have shown that particle size and surface area dose of carbon black and other poorly soluble particles influence the pulmonary inflammation response, considered key in the pathway to particle-induced lung cancer in rats (Schins and Knaapen 2007). Research needs include updating epidemiology cohorts with data on work histories and exposures in relation to particle size and surface area, and recruitment of additional carbon black facilities. The relationship between occupational exposure to carbon black and validated biomarkers of oxidative stress should be examined and exposure–response relationships in humans and rodents quantified, including the role of particle size.

#### Styrene and styrene-7,8-oxide

In 2008, a U.S. National Toxicology Program (NTP) expert panel reviewed styrene, finding limited evidence in humans but sufficient evidence of animal carcinogenicity from multiple studies in mice by multiple routes (Styrene Expert Panel 2008). Epidemiologic studies of styrene in the styrene–butadiene rubber industry have been limited by multiple exposures, a limitation partially addressed by retrospective exposure assessment (Sathiakumar et al. 2005). Studies in the fiberglass boat– building industry have been limited by small size and short duration of exposure (Ruder et al. 2004). Interpretation of the epidemiologic evidence is complicated by findings of higher risk in less-exposed cohorts, variation in high-LHC sites in different studies, and inconsistency in findings for pancreatic cancer. At least 70 publications released since the styrene monograph (IARC 2002) explore various mechanistic aspects of potential carcinogenicity in humans and rodents. Recommendations for new research include pooled analyses of human studies on chromosome aberrations and other genotoxic effects and updating the existing epidemiologic studies with particular attention to the accurate diagnosis and classification of LHCs.

#### Propylene oxide

Since the last IARC review (IARC 1994), only one epidemiologic study of U.S. propylene oxide (PO) manufacturing workers has been published (Olsen et al. 1997); the authors did not find increased mortality due to cancer by duration of exposure with or without latency, nor did they find increased cancer risk by process (PO vs. ethylene oxide). Recent exposure and biomarker studies have shown that PO forms chemically stable hemoglobin and DNA adducts and that concentrations of these adducts are related linearly to air concentrations of PO (Boogaard et al. 1999); in addition, Czene et al. (2002) reported that hemoglobin and DNA adducts and sister chromatic exchanges were increased significantly in workers occupationally exposed to PO. Potential cohorts for future epidemiologic studies exist in a number of industries and countries; occupational study cohorts should include women, if possible, because PO might be a mammary carcinogen (Rudel et al. 2007).

#### Formaldehyde

Formaldehyde has been classified by IARC as a Group 1 carcinogen based on sufficient evidence for nasopharyngeal cancer in humans (Baan et al. 2009; IARC 2006a). Both IARC and the NTP scientific review panel have recently supported a causal relation between formaldehyde and acute myeloid leukemia based on new research findings (Baan et al. 2009; Beane Freeman et al. 2009; Formaldehyde Expert Panel 2009; Zhang et al. 2009); however, more research is needed to elucidate the mechanism by which formaldehyde could cause myeloid leukemia in humans. Mechanisms through which inhaled formaldehyde may cause leukemia should be explored further, including exposure to circulating blood or stem cells in the nose and pathways by which inhaled formaldehyde or formaldehyde-derived intermediates can reach bone marrow or lymphatic tissue. Follow-up of existing occupational cohorts should continue, with registry linkage to identify incident cancers and attention to appropriate classification and grouping of LHCs. Additional studies of the genotoxic and hematologic effects of formaldehyde exposure in occupational cohorts and in experimental animals would be useful, and such studies should incorporate sensitive biological markers of internal dose.

#### Acetaldehyde

Acetaldehyde is the first metabolite of ethanol oxidation. It binds to DNA, forming stable DNA adducts that are observed in alcohol consumers (Seitz and Stickel 2007). Numerous epidemiologic studies in alcohol drinkers with alcohol dehydrogenase (*ALDH2*) deficiency or low aldehyde dehydrogenase (*ADH1B*) activity (Lachenmeier et al. 2009; Salaspuro 2009) provide the most compelling evidence for the carcinogenicity of acetaldehyde. A recent large-scale case–control study reported a multiplicative combined risk for esophageal cancer among alcohol and tobacco consumers who were low *ADH1B* and *ALDH2*-deficient carriers (OR = 382.3; 95% CI, 47.4–3084.9 for those drinking > 30 g/day) (Lee et al. 2008). These studies strongly suggest that acetaldehyde derived from the metabolism of ethanol contributes to upper digestive tract cancers. The accumulated scientific evidence warrants a new evaluation of acetaldehyde by IARC. Exposures to acetaldehyde in occupational settings should be characterized and the potential for conducting epidemiologic studies explored. These studies should consider all potential sources of exposure to acetaldehyde and the extent to which genetic polymorphisms influence carcinogenic risks. Studies in the flavoring industry may be of particular interest.

#### Trichloroethylene (TCE)

Since the IARC review (IARC 1995b), numerous publications have evaluated associations between TCE exposure in humans and cancers at several sites, including kidney and liver cancer, and non-Hodgkin lymphoma (NHL). Meta-analyses would be useful because individual studies have limited statistical power for these relatively uncommon cancer sites. Additional studies of cancer incidence and mortality in new cohorts without multiple solvent exposures (e.g., those using TCE for a final degreasing after assembly-line production of kitchen utensils) would be beneficial. Research is needed to determine which TCE metabolites are the agents of carcinogenesis for specific sites. Studies of effects of TCE exposure on cell-signaling pathways and epigenetic changes induced by TCE and its metabolites would help in determining potential mechanisms of carcinogenicity. TCE is metabolized by the cytochrome P450 (*CYP*) pathway to oxidative metabolites and by the glutathione (GSH) conjugation pathway to genotoxic metabolites; incorporation of data on genetic polymorphisms in glutathione *S*-transferase and *CYP2E1* would be useful in this regard.

#### Tetrachloroethylene (Perc)

Since the IARC review (IARC 1995a), several human epidemiologic studies have reported associations between Perc exposure and esophageal cancer and NHL, with some evidence for breast, urinary bladder, and kidney cancer (Ruder 2006). Although many industries use Perc, the chief venue of Perc exposure is dry-cleaning shops, which generally have < 10 employees (Gold et al. 2008). Further studies in this industry could be facilitated by using exhaled-breath specimens for study inclusion and exposure assessment (McKernan et al. 2008). Two U.S. dry-cleaning cohorts could be pooled for mortality and cancer incidence studies (Blair et al. 2003; Ruder et al. 2001), and additional cohorts of workers outside the United States and Europe should be identified. A major research gap is that mechanisms of carcinogenicity are not characterized sufficiently or tested; studies are needed that evaluate the genotoxic and oxidative potential of alternative metabolic pathways. Last, adequate physiologically based pharmacokinetic (PBPK) models should be developed that allow for prediction of metabolism and difference in metabolism between species for a number of key metabolites to aid in the identification of sensitive subpopulations and target organs for a carcinogenic response.

#### Methylene chloride [dichloromethane (DCM)]

Inhalation exposure to DCM causes lung and liver tumors in mice and mammary tumors in rats (IARC 1999b). Epidemiologic case–control and cohort studies have found positive, but inconsistent, associations for cancers of a number of sites. Based on animal and epidemiologic studies to date, sites of particular interest for future studies include brain, breast, and the lymphohematopoietic system. Available epidemiologic studies of DCM are limited by small numbers of exposed cases, few women enrolled, and poor exposure assessments. The major research need is the identification of new large cohorts with adequate numbers of women and robust exposure assessment using current and retrospective department-specific exposure or biological markers. In addition to identifying larger cohorts of film and textile workers, some potential new occupations include workers in furniture stripping or automobile body repair shops. Urinary DCM has been shown to correlate with air measurements (Imbriani and Ghittori 2005), and studies are needed to develop and evaluate urinary DCM measurements for use in exposure assessment. Recent mechanistic studies have questioned the role of the GSH pathway in toxicity (Landi et al. 2003; Watanabe and Guengerich 2006; Watanabe et al. 2007). DCM has been reported to be mutagenic in bacteria without activation (IARC 1999b). Clearly, research is needed with regard to the metabolites involved and the mechanism of carcinogenicity of DCM-induced rodent tumors, especially in the context of informing human risk. Before accurate PBPK models can be developed, the metabolism and metabolites responsible for toxicity at specific targets should be investigated.

#### Chloroform (trichloromethane)

Chloroform causes cancer in rats and mice, most likely through a mechanism involving cytotoxicity (Schoeny et al. 2006), and there is weak evidence for the genotoxicity of chloroform (IARC 1999a). Exposure to chloroform is primarily through drinking water and swimming pool water; thus, the epidemiology is based on exposure to this complex mixture and not to chloroform per se. Since the last IARC evaluation (IARC 1999a), several epidemiologic studies have been published on the association between exposure to chloroform in disinfection by-products (DBPs) and risk of bladder cancer, including a pooled analysis of previous case–control studies (Villanueva et al. 2004) and a new case–control study from Spain (Villanueva et al. 2007). However, drinking water with high levels of chloroform also contains high levels of other trihalomethanes (THMs) and other DBPs, and bladder cancer associated with drinking water may result from dermal/inhalation exposure to the brominated THMs or DBPs other than chloroform. Future IARC evaluations should address the entire group of DBPs in drinking water. Exposures to chloroform and other DBPs may be higher from showering, bathing, or swimming than from oral exposure to drinking water. Other THMs/DBPs should be evaluated for biological effects in rodents via the dermal and/or inhalation route. Epidemiologic case–control studies should incorporate information on route of exposure and detailed DBP exposure assessment, as well as pooling information from multiple studies and countries, where feasible. Epidemiologic studies are warranted for high-exposure groups such as competitive swimmers and indoor pool attendants/lifeguards. There should also be follow-up of cohorts of medical personnel exposed to chloroform when chloroform was used as an anesthetic gas.

#### PCBs

Identifying research gaps for PCBs is considerably more difficult, because a large volume of epidemiologic and mechanistic data has been published since the last IARC evaluation (IARC 1987). Moreover, mixtures of PCBs associated with occupational and environmental exposure have changed over time and vary across the occupational and population groups studied. In addition, environmental and metabolic processes substantially alter the composition of PCB mixtures in the environment and in the body. As a result, residual PCBs in the environment involve altered mixtures differing in composition—and possibly more toxic and persistent—than the mixtures that were used commercially (Cogliano 1998). Among most occupational cohorts, dermal and airborne exposures predominate, whereas among the general population, dietary exposures are generally most significant. Although most studies of highly exposed occupational cohorts find cancer excesses for specific cancer sites, the sites involved have been quite variable. Associations between NHL and levels of certain PCB congeners in serum have been reported in several cohort and case–control studies (Engel et al. 2007; Rothman et al. 1997), whereas studies of serum levels of PCBs and breast cancer have been inconsistent, although largely negative (Ward et al. 2000). Additional studies within highly exposed populations, including an in-progress cancer incidence study within the large (> 26,000 workers) NIOSH cohort (Prince et al. 2006; Ruder et al. 2006), nested case–control studies in this cohort and/or occupational cohorts in other countries, and analysis of PCB blood levels in cases and controls, might be informative. Mechanisms of genotoxicity/carcinogenicity for PCBs appear to involve ROS, oxidative stress, oxidative DNA damage, and formation of DNA adducts (Jeong et al. 2008; Ludewig et al. 2008). More research is needed on these mechanisms and on cell proliferation, which could also play an important role in the induction of mutations and subsequent carcinogenicity.

#### Di(2-ethylhexyl) phthalate (DEHP)

Although extensive human exposure to DEHP occurs through its use as a plasticizer of polyvinyl chloride (PVC), definitive epidemiologic studies are not available because of the difficulty in identifying highly exposed workers in retrospective cohort or case–control studies. Since the previous monograph review, which concluded that liver cancer in animals resulted from induction of peroxisome proliferator-activated receptor-α (PPARα) and that peroxisome proliferation activation was not relevant to humans (IARC 2000), several lines of evidence have suggested that DEHP may have multiple mechanisms of carcinogenesis, such as induction of cell proliferation, decreased apoptosis, and oxidative DNA damage, some of which might be relevant to humans (Rusyn et al. 2006). The hypothesized PPARα mode of action has also been questioned (Guyton et al. 2009). A study of DEHP-induced tumorigenesis in wild-type and PPARα-null mice found that the incidence of liver tumor in PPARα-null mice exposed to 0.05% DEHP was higher (25.8%) than that in similarly exposed wild-type mice (10.0%) (Ito et al. 2007). Microarray profile studies found that patterns of up- or down-regulated genes are quite different in hepatocellular adenoma tissues of wild-type and PPARα-null mice exposed to DEHP (Takashima et al. 2008). Animal studies have also suggested additional target organs in rats [pancreatic acinar-cell adenoma (David et al. 2000) and testicular Leydig cell tumors (Voss et al. 2005)]. Future studies in mouse models using hPPARα^TetOff^ (which expresses the human receptor only in liver) or hPPARα^PAC^ (which expresses the human receptor in liver, kidney, heart, intestine, and brown adipose tissues) may elucidate the role of human PPARα in DEHP carcinogenesis. Further characterization of DEHP exposures in industry is needed and could be carried out in established cohorts in PVC-processing factories using mono-2-ethylhexyl phthalate and mono(2-ethyl-5-carboxypentyl) phthalate as sensitive and specific biomarkers of DEHP exposure.

#### Atrazine

Schoeny et al. (2006) reported that atrazine caused mammary gland tumors in Sprague-Dawley rats through accelerated aging within the brain–pituitary–ovarian axis (i.e., constant estrus); however, they found that it was not carcinogenic in F344 rats or via the diet in CD-1 mice, but it did cause lymphomas via intraperitoneal injection in CD-1 mice. Although the mechanism by which atrazine causes mammary tumors in Sprague-Dawley rats may not be relevant to humans (Schoeny et al. 2006), additional studies would help to clarify the situation. For example, does atrazine interfere with the hypothalamic–pituitary–ovarian axis or alter the secretion of luteinizing hormone and prolactin in humans? More extensive microarray and proteomic studies in rodents and humans would help to characterize the pathways disrupted by atrazine. Studies should also investigate the ability of atrazine to alter immune function and aromatase in species relevant to humans, as well as in human molecular epidemiology studies. Several studies have found nonsignificant associations between atrazine exposure and NHL; for example, a study of 36,513 atrazine-exposed pesticide applicators in the U.S. Agricultural Health Study (AHS) demonstrated nonsignificant excesses of lung cancer, bladder cancer, NHL, and multiple myeloma (Rusiecki et al. 2004). Follow-up of the AHS cohort through 2006 is now under way and, along with analysis of biomarkers among corn farmers and similar studies in atrazine-exposed women (Bakke et al. 2008; Vermeulen et al. 2005), could shed light on the effects of atrazine.

#### Shift work

Excess incidence of breast cancer has been observed consistently in studies of women with prolonged exposure to shift work involving exposure to light at night (Kolstad 2008; Stevens 2009). Research needs in this area include *a*) a better definition of what is meant by shift work and related exposure metrics; *b*) studies of markers of circadian disruption in non–day workers; *c*) better descriptions of controls and their exposure to light at night; and *d*) investigation of the effect of variations in expression of circadian genes on cancer in shift workers. An emerging area of interest is the relative toxicity of occupational chemical exposure depending on time of day of that exposure. The marked circadian variations in cell division and DNA repair during the daily cycle are controlled by the circadian genes (Haus and Smolensky 2006; Stevens et al. 2007). Therefore, non–day workers may have very different susceptibility to occupational exposures compared with day workers. Studies are also needed to determine if shift work is associated with other cancers, especially hormonally related cancers, and prostate cancer in particular. If further experimental and epidemiologic evidence confirms a causal association between exposure to light at night and breast cancer, it will be important to develop interventions to reduce the risk.

## Conclusions

Research gaps and opportunities have been identified that can help to resolve uncertainties regarding the carcinogenicity in humans of a number of important IARC-classified agents. We hope that this process will lead to well-planned epidemiologic and mechanistic studies for these agents, as well as renewed interest and funding for studies of agents for which there are substantial or widespread occupational and environmental exposures.

Several important scientific developments are likely to increase the challenges and complexities of carcinogen testing and evaluation in the future. Use of omics techniques will accelerate the understanding of the cellular and molecular basis for biological responses to environmental and occupational exposures, and high-throughput technologies will increase the number of agents that can be tested. The important role of organ and organism-level responses such as inflammation, immunomodulation, and hormonal influences, as well as interindividual variation in susceptibility and genetic repair in the carcinogenic process, are increasingly understood. Therefore, the science of carcinogen testing and evaluation must be increasingly multidisciplinary, examining biologic responses from the molecular to the organism, and using test systems and approaches that capture multiple mechanisms and end points.

Most carcinogenic mechanisms are not simple, and evidence is often too limited to conclude lack of relevance to humans. When evidence regarding mechanism is considered in the upgrading or downgrading of carcinogens, it should be evaluated with the same rigor as traditional epidemiologic and bioassay data [see, for example, the IARC preamble (IARC 2006e) with regard to epidemiologic studies, including types of studies to be considered, quality of studies, role of meta- and pooled analyses, and criteria for causality]. Epidemiologic studies will be particularly critical in evaluating the relationship between intermediate biomarkers and cancer risk in occupational groups or in the general population and in investigating genetic susceptibility factors. In the rush to embrace new biotechnologies in epidemiology, we must not lose sight of the tremendous gains in knowledge that have accrued from conventional epidemiologic occupational cohort and case–control studies. We encourage investigators to continue to search for study populations in which the linkage of work history information and mortality or cancer incidence data can be informative about the cancer risks of workers with different job or industry titles or different exposure histories. We also encourage release (in deidentified form) of completed epidemiologic studies for reanalysis, as is commonly done with government-funded studies in the United States.

In this review we discuss only a small fraction of potentially carcinogenic agents—generally those for which there is substantial evidence for animal carcinogenicity but as yet inconclusive evidence of human carcinogenicity. For most other agents, there exists little or no evidence by which to evaluate animal or human carcinogenicity because neither adequately designed animal bioassays nor human studies have been done. However, even the modest research agenda outlined here will be difficult to achieve given current trends. Although objective evaluation of trends in research funding and productivity related to environmental/occupational risk factors for cancer is difficult, there is a general sense that funding for occupational cancer research has declined over the past two decades and that fewer and fewer epidemiologists, exposure assessment experts, and toxicologists are attracted to careers in this area. A more formal evaluation of these trends could consider what measures could encourage *a*) renewed interest in this field; *b*) training, career, and funding opportunities; and *c*) advances in addressing legal–ethical barriers to accessing worksites, study populations, and personal/medical data. If measures are not taken to stem declines in this area of research, we will continue to have an extremely limited epidemiologic knowledge base for the evaluation of potential carcinogens.
